# Advancing precision risk stratification in adult diffuse gliomas through DSC-MRI-based habitat analysis

**DOI:** 10.1186/s12880-026-02509-7

**Published:** 2026-06-23

**Authors:** Weiqiang Liang, Jie Zhou, Yi Huang, Pingyue Zou, Dan Xu, Haibo Xu

**Affiliations:** 1 Department of Radiology, Zhongnan Hospital of Wuhan University, 169 Donghu Road, Wuhan, 430071 China; 2 Department of Nuclear Medicine, Zhongnan Hospital of Wuhan University, Wuhan University, Wuhan, 430071 China; 3 Hubei Provincial Engineering Research Center of Multimodal Medical Imaging Technology and Clinical Application, Wuhan, 430071 China

**Keywords:** Glioma, Dynamic susceptibility contrast MRI, Radiomics, Habitat analysis, High-risk molecular subtypes

## Abstract

**Objectives:**

Diffuse gliomas exhibit substantial molecular and spatial heterogeneity. This study aimed to evaluate the ability of habitat-based radiomics models derived from dynamic susceptibility contrast MRI (DSC-MRI) and conventional MRI to identify aggressive diffuse glioma phenotypes associated with integrated histologic-molecular risk.

**Methods:**

This retrospective study included 197 adult patients with histopathologically confirmed diffuse gliomas. Multiparametric MRI data were preprocessed and segmented into tumor and peritumoral edema. K-means clustering was used to identify imaging-defined habitats reflecting spatial hemodynamic heterogeneity. A total of 855 radiomic features were extracted from each habitat and reduced through a sequential selection process involving univariate statistical testing, correlation filtering, recursive feature elimination, and LASSO regression. Random forest classifiers were developed to predict high-risk molecular subtypes, including IDH wildtype and other aggressive genetic alterations, and validated in internal held-out testing cohort.

**Results:**

Habitat-based models significantly outperformed whole-region analyses (AUC 0.949 (0.858, 0.989) vs. 0.931(0.833, 0.980), *p* = 0.013). Crucially, models derived from hemodynamic features (CBF + MTT) demonstrated superior predictive accuracy compared to conventional anatomical sequences (T1C+T2FLAIR) in both tumor (AUC 0.944 (0.852, 0.987) vs. 0.895 (0.787, 0.960)) and edema habitats (AUC 0.932 (0.835, 0.981) vs. 0.819(0.698, 0.907)). The optimal model relied solely on hemodynamic features from combined habitats (AUC 0.949 (0.858, 0.989)). Multimodal fusion failed to improve performance, suggesting that hemodynamic parameters may provide the most discriminative imaging information.

**Conclusion:**

DSC-MRI-based habitat analysis provides significant value over conventional imaging by resolving perfusion heterogeneity. These findings highlight that hemodynamic features serve as a promising tool for prediction of integrated histologic-molecular risk status of adult diffuse gliomas, potentially serving as a promising imaging biomarker for preoperative assessment.

**Supplementary Information:**

The online version contains supplementary material available at https://doi.org/10.1186/s12880-026-02509-7.

## Highlights

Diffuse gliomas present significant diagnostic challenges due to heterogeneity.

Hemodynamic habitat analysis significantly outperforms conventional anatomical imaging.

This approach provides a key non-invasive tool for prediction of integrated molecular risk status.

**Supplementary Information:**

The online version contains supplementary material available at https://doi.org/10.1186/s12880-026-02509-7.

## Introduction

Diffuse gliomas are the most common primary malignant tumors of the adult central nervous system (CNS), characterized by pronounced heterogeneity, infiltrative growth, extensive peritumoral edema, and disruption of the blood–brain barrier, often resulting in poor prognosis [1]. According to the 2016 and 2021 classifications of gliomas by the World Health Organization (WHO), molecular subtypes and histological grading play a crucial role in determining prognosis and guiding therapeutic strategies [2–4]. Although conventional clinical assessments—such as tissue biopsy and molecular marker analysis—provide important diagnostic information, their utility is limited by tumor heterogeneity, sampling bias, and the invasive nature of the procedures. These challenges have prompted increasing interest in developing non-invasive and accurate imaging approaches to evaluate the molecular characteristics and predict the prognosis of gliomas.

Magnetic resonance imaging (MRI) is the standard imaging modality for glioma evaluation and is widely used for tumor detection, grading, and longitudinal monitoring. While conventional MRI (e.g., T1C, T2FLAIR) is effective for structural delineation, it fundamentally relies on blood-brain barrier disruption and morphological changes. Consequently, it often fails to resolve the underlying functional heterogeneity—such as aberrant angiogenesis and hypoxia—which are hallmarks of high-risk molecular subtypes. Dynamic susceptibility contrast MRI (DSC-MRI), which enables the assessment of tumor perfusion and hemodynamic characteristics, offers a promising avenue for capturing the biological heterogeneity of gliomas beyond conventional imaging [5–8].

In recent years, radiomics has emerged as a powerful tool for quantitatively analyzing medical images [9]. By extracting a large number of high-dimensional features from imaging data, radiomics enables the characterization of tumor heterogeneity and biological behavior, including angiogenesis, cellularity, and microstructural complexity [10]. When combined with deep learning techniques, radiomics allows for automated feature extraction and predictive modeling, offering a non-invasive strategy for tumor grading and prognostic evaluation [11]. Several studies have demonstrated that radiomic features are significantly associated with molecular alterations, such as IDH mutation and 1p/19q codeletion, as well as tumor recurrence risk [12–15].

However, most radiomics studies adopt a “whole-tumor” approach, extracting features from the entire lesion volume. This global averaging strategy inevitably dilutes critical local signals, potentially masking small but aggressive sub-clones driving poor prognosis. To address this, “habitat imaging” has emerged as a strategy to decompose the tumor into biologically distinct subregions. By mapping spatial variations in hemodynamics, we can potentially identify “high-risk niches” that are invisible to global analysis [16–18].

This study aims to establish a proof-of-concept framework for assessing the value of hemodynamic habitat analysis in predicting integrated molecular risk status. We applied unsupervised clustering to segment tumors into imaging-defined habitats based on DSC-MRI perfusion patterns and anatomical signals. We hypothesized that hemodynamic habitats derived from DSC-MRI capture unique microenvironmental heterogeneity, thereby outperforming conventional anatomical models and global radiomics analyses in stratifying high-risk patients.

## Methods

### Patient cohort

This retrospective study was approved by the Ethics Committee of our institution (Approval No. 2020109). Informed consent was waived due to the retrospective nature of the study.

We retrospectively reviewed data from adult patients who were diagnosed with gliomas at the Department of Neurosurgery of our hospital between January 2020 and December 2024. A total of 255 patients were initially screened, and 58 were excluded (Supplementary Fig. 1). The inclusion criteria were as follows: (1) histopathologically confirmed adult diffuse gliomas in accordance with the 2021 WHO classification of CNS tumors; (2) availability of complete preoperative brain MRI scans, clinical data, and pathological reports. Exclusion criteria were: (1) prior treatment including biopsy, radiotherapy, or chemotherapy; (2) incomplete imaging or clinical data; (3) poor MRI image quality.

All eligible patients were randomly divided into a training cohort (*n* = 138) and a testing cohort (*n* = 59) in a 7:3 ratio. The clinical and pathological characteristics of both cohorts are summarized in Table 1.

**Table 1:** Clinical characteristics of the training cohort and validation cohort

Characteristics	Training dataset (*n* = 138)			Test dataset (*n* = 59)			
	High-risk molecular subtypes(*n* = 88)	Non-high-risk molecular subtypes(*n* = 50)	*p*	High-risk molecular subtypes(*n* = 36)	Non-high-risk molecular subtypes(*n* = 23)	*p*	*p* ^*^
Age (years)	54.9 ± 12.8	45.6 ± 14.4	< 0.001	58.0 ± 10.9	51.6 ± 10.2	0.029	0.094
Gender			0.187			0.571	0.217
Male	49	22		23	13		
Female	39	28		13	10		
Grade			< 0.001			< 0.001	0.174
2,3	8	50		8	23		
4	80	0		28	0		
Ki-67 (%)	30.0(25.0)	6.0(11.5)	< 0.001	30.0(20.0)	5.0(7.0)	< 0.001	0.941

Age is presented as mean ± standard deviation; Ki-67 is shown as median (interquartile range). All other values represent the number of individuals. *p*, significance of difference between high- and low-risk molecular subtypes; p^*^, significance of difference between training and validation sets

### Molecular subtype assessment

Tumor tissues obtained during surgery were subjected to Sanger sequencing and next-generation sequencing to assess molecular alterations, including IDH1/2 and TERT mutations, EGFR amplification, + 7/-10 copy number variations, homozygous deletion of CDKN2A/B, and 1p/19q codeletion. Ki-67 immunohistochemistry was performed to evaluate tumor cell proliferation. The Ki-67 labeling index was defined as the percentage of Ki-67–positive nuclei among all tumor cells. Molecular subtype classification followed the high-risk molecular workflow based on the 2021 WHO CNS tumor classification, with reference to Yang et al. [19] (Supplementary Fig. 2, Supplementary Table 1).

### Image acquisition and post-processing

All MRI examinations were conducted using a 3.0T scanner (uMR 790, United Imaging Healthcare, Shanghai, China) equipped with a 24-channel head coil. The imaging protocol included 3D-T1WI, T2WI, 3D T2FLAIR, 3D T1C, and DSC-MRI. DSC-MRI was performed during intravenous administration of a standard dose (0.1 mmol/kg) of gadolinium-based contrast agent. Detailed acquisition parameters are provided in Supplementary Table 2.

### Image preprocessing

To ensure reproducibility and minimize center-specific bias, all image preprocessing pipelines strictly adhered to the Image Biomarker Standardization Initiative (IBSI) guidelines. All imaging data were preprocessed using ANTs (version 2.5.3, University of Pennsylvania, https://github.com/ANTsX/ANTs) and FSL (version 6.0.1, University of Oxford, UK, http://www.fmrib.ox). T1C and T2FLAIR images underwent N4 bias field correction and non-local means denoising. Motion correction of DSC-MRI time series was performed using FSL’s mcflirt, followed by calculation of perfusion maps (e.g., cerebral blood flow (CBF), mean transit time (MTT)). All images were linearly registered to the MNI152 standard space using FSL’s flirt function and skull-stripped using the bet tool. All images were resampled to a resolution of 1 × 1 × 1 mm³.

### DSC-MRI postprocessing

All DSC-MRI post-processing was performed using MATLAB (R2021a, MathWorks, Natick, MA, USA; https://www.mathworks.com). Raw DSC-MRI signals were normalized to baseline and converted into transverse relaxation rate changes (ΔR2*) using an exponential decay model to quantify dynamic contrast concentrations. A single dose of 0.1 mmol/kg Gadodiamide injection (0.5 mmol/mL) was injected intravenously at 4.5 mL/s via a 20G catheter; no preload bolus was administered. Arterial input functions (AIFs) were identified via a semi-automated approach from the M1 segment of the middle cerebral artery (MCA) combining early time-slope analysis and morphological enhancement, with validation based on their time–concentration curves. The tissue residue function was estimated using singular value decomposition (SVD) deconvolution, from which CBF, cerebral blood volume (CBV), and MTT were derived. Boxerman-Schmainda-Weisskoff (BSW) leakage correction was applied during DSC-MRI postprocessing to partially mitigate T1 leakage effects associated with contrast extravasation. Further methodological details are available in the Supplementary materials.

### Image segmentation

The regions of interest (ROIs) included the entire tumor (enhancing component and central necrosis) and peritumoral edema. Automated lesion segmentation was performed using Segmentation services available on the ONCOhabitats platform, which employs a 3D convolutional neural network classifier based on the U-Net architecture to delineate tumor and peritumoral regions [20, 21]. Segmentation results were independently reviewed and corrected by two neuroradiologists blinded to clinical, pathological, and molecular information, using ITK-SNAP software (version 3.8.0, http://itksnap.org). Final segmentations were determined by consensus and used as ground truth.

### Habitat mapping

Using multiparametric MRI data (T1C, T2FLAIR, CBF, MTT), voxel-wise intensity values and local radiomic features (mean, standard deviation, range, and interquartile range within a 3 × 3 × 3 voxel neighborhood) were extracted from tumor and edema regions to characterize spatial heterogeneity. K-means clustering was applied across the training partition to group voxels with similar intensity and radiomic patterns. The optimal number of clusters (k = 2–10) was determined by performing 10 iterations per k and calculating the mean Calinski-Harabasz score. The final clustering was based on 50 repetitions with the optimal k, selecting the result with the lowest inertia. Each tumor was thus divided into subregions (habitats) representing distinct tumor microenvironments.

### Feature extraction and data partitioning

Before any image processing or feature engineering, the entire patient cohort (*N* = 197) was randomly stratified into a training cohort (70%, *n* = 138) and a held-out internal verification cohort (30%, *n* = 59) based on patient IDs to prevent forward-looking data leakage. Radiomics features were extracted from multiparametric MRI (T1C, T2FLAIR, CBF, MTT) data using the PyRadiomics software package (v 3.0.1). In compliance with the IBSI guidelines, images were resampled to 1 × 1 × 1 mm³ isotropic voxels using B-spline interpolation, and intensity histogram discretization was performed with a fixed bin width (FBW = 5) for parametric maps. A total of 855 radiomics features were extracted from each region of interest (including the tumor core, peritumoral edema, and defined habitats). These features covered the following categories: (1) shape features (*n* = 14); (2) first-order statistical features (*n* = 18); (3) texture features, including gray-level co-occurrence matrix (GLCM, *n* = 22), gray-level run-length matrix (GLRLM, *n* = 16), gray-level size-zone matrix (GLSZM, *n* = 16), gray-level dependence matrix (GLDM, *n* = 14), and neighborhood gray-tone difference matrix (NGTDM, *n* = 5); and (4) wavelet-transformed features (*n* = 750).

Before feature selection, the entire patient cohort was randomly stratified into a training cohort (70%) and an independent testing cohort (30%) based on patient IDs.

### Feature selection and model development

A rigorous and sequentially nested feature selection pipeline was implemented within the training cohort to identify the most robust and discriminative radiomics signature. First, Z-score standardization parameters were calculated on the training cohort and subsequently applied to transform the verification cohort. Features with low variability were removed via variance thresholding (threshold = 0.01). Subsequently, biologically relevant features showing significant intergroup differences were identified using independent two-sample t-tests with Benjamini-Hochberg false discovery rate (FDR) correction (adjusted *p* < 0.01). To address multicollinearity, a Pearson correlation coefficient matrix was calculated, and for feature pairs with high correlation (|r| > 0.9), one feature was removed to reduce redundancy. Finally, the Least Absolute Shrinkage and Selection Operator (Lasso) regression with 10-fold cross-validation was employed to select the optimal feature subset. The L1 penalty term was tuned to minimize binomial deviance, effectively shrinking the coefficients of redundant variables to zero.

**Fig. 1:**
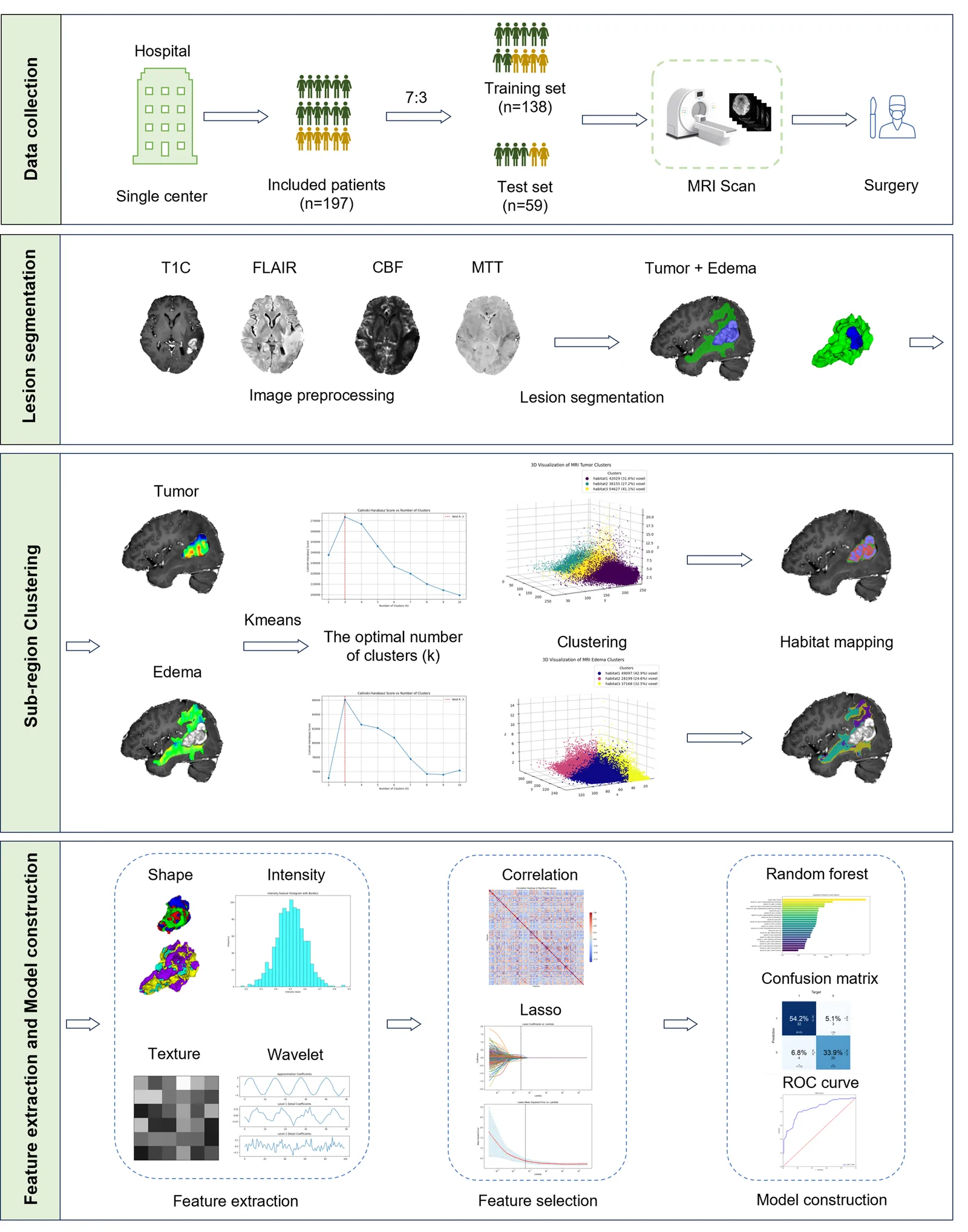
Flowchart of the radiomics workflow

Using the selected features, Random Forest (RF) classifiers were constructed to predict high-risk molecular subtypes. Hyperparameters were optimized via grid search on the training set (n_estimators = 100, max_depth = None, min_samples_split = 2, min_samples_leaf = 1, max_features = ‘sqrt’, random_state = 42). A 5-fold cross-validation was performed on the training set to assess model stability. Models were developed separately for different tumor compartments and input modalities. Model performance was evaluated using the area under the receiver operating characteristic curve (AUC), sensitivity, and specificity. Model calibration was assessed via the Brier score. All statistical analyses and modeling were implemented using Python (Scikit-learn v1.0.2) with a fixed random seed (42) for reproducibility. The entire workflow of the study is illustrated in Fig. 1.

### Statistical analysis

All statistical analyses were conducted using Python (3.12.7, https://www.python.org/) and MedCalc (18.2.1; http://www.medcalc.org/) software. Continuous variables were compared using the Wilcoxon rank-sum test, and categorical variables using the chi-square test. The AUC and corresponding 95% confidence intervals (CI) were used to assess model performance in predicting high-risk molecular subtypes. AUC comparisons between models were performed using DeLong’s test. Decision Curve Analysis (DCA) was performed to evaluate the clinical net benefit of the proposed models across a range of threshold probabilities. A p-value < 0.05 was considered statistically significant.

## Results

### Patient clinical characteristics

Table 1 summarizes the baseline clinical and pathological characteristics. A total of 197 patients were enrolled (training cohort: *n* = 138; independent testing cohort: *n* = 59). Patients with high-risk molecular subtypes were significantly older compared to the non-high-risk group in both cohorts (training: *p* < 0.001; testing: *p* = 0.029). Sex distribution showed no significant bias (*p* > 0.05). Notably, higher pathological grades and Ki-67 proliferation indices were strongly associated with the high-risk group (all *p* < 0.001), reflecting the aggressive biological nature of these tumors.

### Habitat feature distribution and visualization

K-means clustering was applied to spatially subdivide the tumor and peritumoral edema regions. Systematic evaluation using the Calinski-Harabasz criterion revealed that a cluster number of three achieved the highest score, indicating an optimal balance between maximizing inter-cluster separation and minimizing intra-cluster heterogeneity (Supplementary Fig. 3). The resulting habitats corresponded to distinct biological subregions within the tumor.

### Performance evaluation of different predictive models

Multiple radiomics models were constructed based on different imaging combinations—T1C+T2FLAIR, CBF + MTT, and a multi-modal fusion of both (T1C+T2FLAIR + CBF+MTT)—across various regions of interest, including the whole tumor (WT), whole edema (WE), tumor habitats (TH), and edema habitats (EH). The predictive performance for high-risk molecular subtypes varied depending on imaging modality and region of interest.

Overall, habitat-based models (TH and EH) demonstrated superior performance (AUC = 0.819 (0.698, 0.907) -0.949 (0.858, 0.989)) compared with models based on whole tumor or edema regions (AUC = 0.808 (0.684, 0.899)–0.931 (0.833, 0.980)), with a statistically significant difference (*p* = 0.013; Supplementary Fig. 4).

**Fig. 2:**
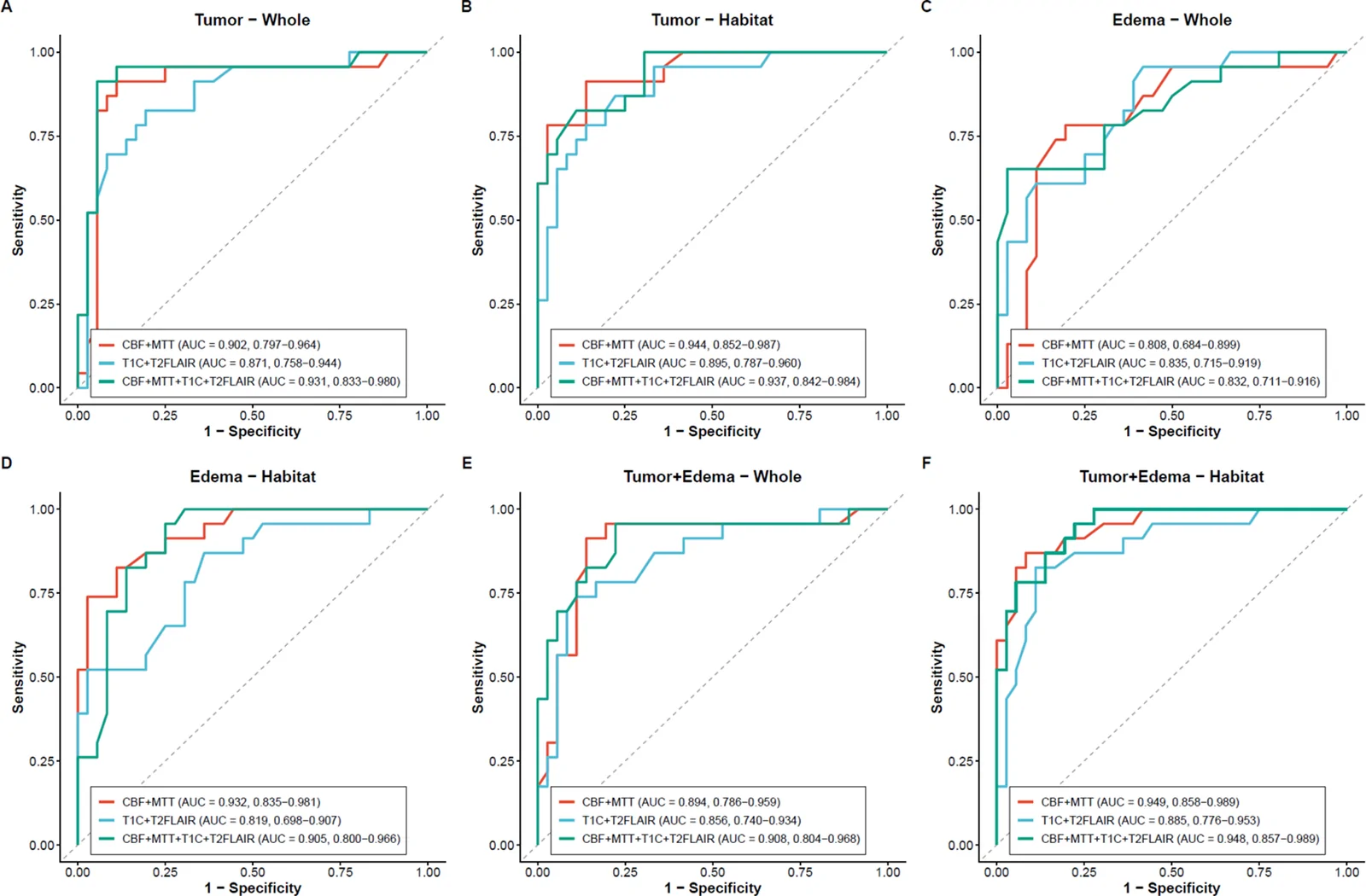
ROC curves of different predictive models on the validation set. The three plots on the left represent models based on the entire region, while the three on the right represent habitat-based models. Among all models, the habitat-based model incorporating CBF and MTT parametric maps demonstrated the highest predictive performance. CBF, cerebral blood flow; MTT, mean transit time; AUC, the area under the receiver operating characteristic curve

**Fig. 3:**
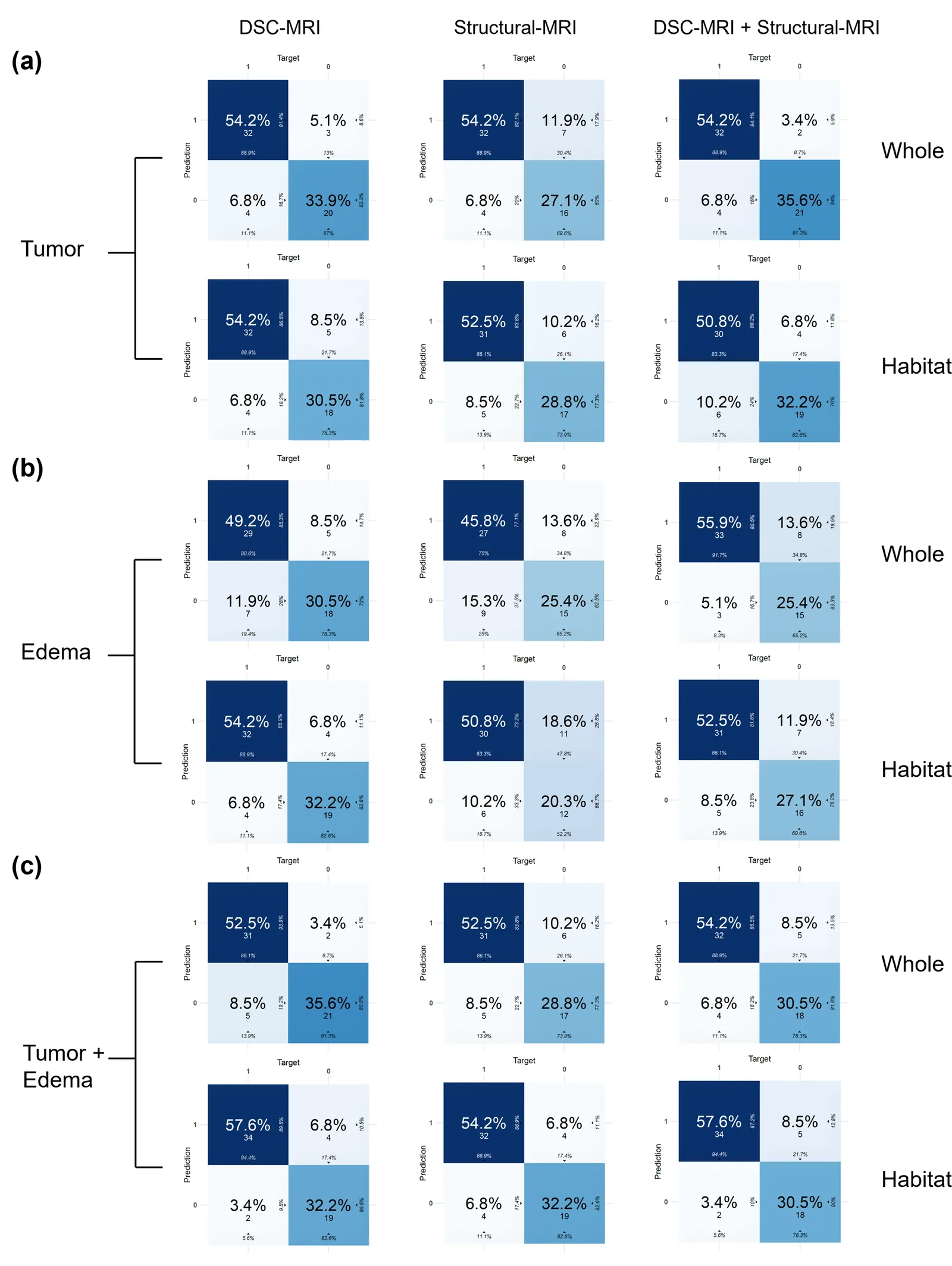
Confusion matrices of different predictive models. Panels **a–c** correspond to models based on tumor, edema, and tumor + edema regions, respectively. The top row shows results from whole-region models, while the bottom row presents habitat-based models. The columns from left to right represent models built on DSC-MRI, structural MRI, and a combination of both modalities

**Fig. 4:**
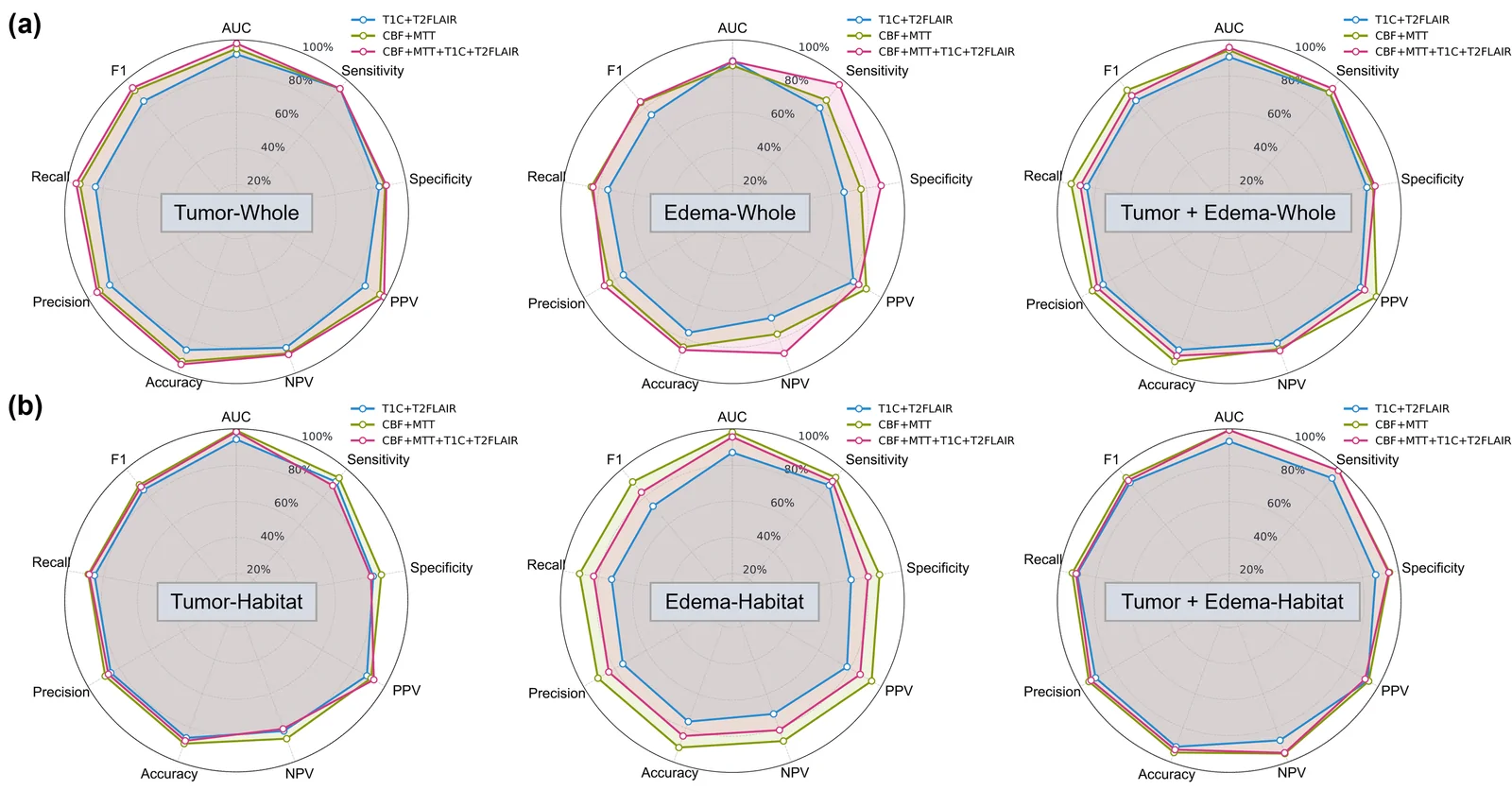
Radar charts of different predictive models. The top row **(a)** displays whole-region models, while the bottom row **(b)** presents habitat-based models

When comparing imaging modalities, models based on hemodynamic parameters (CBF + MTT) outperformed those using anatomical sequences (T1C+T2FLAIR), particularly in TH and EH regions (TH: AUC = 0.944 (0.852, 0.987) vs. 0.895 (0.787, 0.960); EH: AUC = 0.932 (0.835, 0.981) vs. 0.819 (0.698, 0.907))(Fig. 2, Supplementary Table 3). Confusion matrix analysis further confirmed this trend: CBF + MTT models exhibited higher accuracy for predicting positive cases (Target = 1) and fewer misclassifications in negative cases (Target = 0), compared with T1C+T2FLAIR models **(**Fig. 3). Radar plots showed that CBF + MTT-based models outperformed T1C+T2FLAIR models across key metrics including F1-score, recall, and precision in all regions of interest (Fig. 4). Notably, the model trained on TH + EH using CBF + MTT achieved the highest predictive performance (AUC = 0.949 (0.858, 0.989)). However, the addition of multi-modal feature fusion (T1C+T2FLAIR + CBF+MTT) did not yield a significant improvement in overall model performance.

DCA demonstrated that the habitat-based hemodynamic model achieved the highest net benefit across a wide range of threshold probabilities, significantly outperforming the conventional whole-tumor anatomical model. Further analysis indicated that the combined Tumor and Edema model based on hemodynamic features yielded the most robust net benefit, outperforming models based on single regions or anatomical sequences alone (Supplementary Fig. 5).

## Discussion

This study integrated multi-parametric MRI-based radiomic features with spatial heterogeneity analysis of the tumor microenvironment to develop predictive models based on DSC-MRI hemodynamic parameters and conventional anatomical MRI for the noninvasive identification of diffuse glioma phenotypes associated with integrated histologic-molecular risk characteristics. Our findings demonstrate that habitat-based models derived from DSC-MRI effectively capture the biological heterogeneity within tumors and peritumoral regions, providing a novel imaging perspective for non-invasive glioma risk characterization.

First, using the K-means clustering algorithm, we partitioned tumor and peritumoral edema regions into subregions characterized by hemodynamic heterogeneity, thereby overcoming the limitations of conventional radiomic analyses that rely on whole-tumor or whole-edema feature extraction. The three imaging-defined habitats identified by the Calinski-Harabasz criterion may reflect differing microenvironmental characteristics related to vascularity, perfusion heterogeneity, infiltrative behavior, or cellular density. Notably, habitat-based models (TH and EH) showed significantly better predictive performance compared to whole-tumor or whole-edema models, suggesting that conventional global feature extraction may obscure critical spatial heterogeneity, potentially omitting biologically meaningful information. Habitat segmentation enhances the spatial continuity of the tumor microenvironment by leveraging local radiomic features, thereby more precisely capturing the biological characteristics at the invasive front of the tumor [18, 22].

Second, our findings underscore the unique value of hemodynamic parameters in identifying diffuse glioma phenotypes associated with integrated histologic-molecular risk. CBF and MTT features derived from DSC-MRI demonstrated superior predictive performance over traditional anatomical features (T1C/T2FLAIR), particularly in the TH region (AUC = 0.944 vs. 0.895). This superiority is closely linked to the pathophysiological mechanisms underlying high-risk gliomas, which often involve EGFR amplification and overexpression of vascular endothelial growth factor (VEGF), resulting in aberrant angiogenesis and increased perfusion heterogeneity [23–25]. CBF may directly reflect microvascular density, while prolonged MTT may indicate vascular disorganization and impaired perfusion efficiency [26–28]. Biologically, the habitat characterized by elevated CBF and shortened MTT may reflect regions of increased vascularity and perfusion heterogeneity that are commonly observed in biologically aggressive tumor subregions. Conversely, habitats within the peritumoral edema showing restricted perfusion but prolonged transit time may reflect heterogeneous tissue characteristics related to edema, vascular alterations, or tumor infiltration. However, direct histopathological validation was not available in the present study [18, 29, 30].

Interestingly, the integration of anatomical sequences with hemodynamic parameters did not significantly improve model performance, challenging the conventional assumption that multimodal models necessarily outperform unimodal ones. We propose several potential explanations: (1) Feature redundancy—habitat-based feature extraction already incorporates spatial texture information through local radiomic statistics, potentially diminishing the incremental value of T1C/T2FLAIR; (2) Signal sensitivity differences—DSC-MRI may already capture key biological information also reflected in anatomical sequences (e.g., high CBF regions may overlap with contrast-enhanced areas), leading to feature collinearity; (3) Model complexity constraints—random forest classifiers may have limited capacity to exploit high-dimensional synergistic interactions among multimodal features [31, 32]. These findings suggest that in the context of spatial heterogeneity analysis, hemodynamic features may already encompass critical discriminative information for identifying high-risk subtypes, and indiscriminate inclusion of additional modalities may introduce noise. Future research should explore optimized feature-level or decision-level fusion strategies and consider deep learning architectures with attention mechanisms to enhance multimodal integration.

Moreover, the strong predictive performance observed in the peritumoral edema habitat suggests that ostensibly “non-tumoral” regions may harbor meaningful molecular information. This aligns with emerging evidence of tumor cell infiltration and immune microenvironment remodeling within peritumoral edema, further supporting the rationale for including these regions in risk characterization analyses [33–36].

From a translational perspective, the proposed noninvasive approach may provide complementary imaging information for preoperative risk assessment of diffuse gliomas, thereby potentially assisting personalized treatment planning. For instance, patients identified as having elevated imaging-derived risk may potentially benefit from closer clinical surveillance and individualized treatment planning. However, prospective multicenter validation remains necessary before routine clinical implementation. The proposed framework may provide complementary imaging information for preoperative risk assessment. However, prospective multicenter validation and integration with clinical baseline variables remain necessary before clinical implementation.

Nonetheless, several limitations warrant consideration. First, this retrospective study was conducted at a single center using a single scanner, and while an internal held-out testing cohort was used, external generalizability is limited, requiring future multicenter validation. Second, the high-risk molecular subtype endpoint demonstrated strong overlap with WHO grade 4 tumors, so part of the predictive performance may reflect imaging correlates of aggressive histopathology rather than independent molecular features. Therefore, the proposed framework should be interpreted primarily as a tool for identifying aggressive diffuse glioma phenotypes enriched for molecular risk characteristics rather than as a pure molecular classification model. Third, DSC-MRI has inherent technical limitations, including relatively low native through-plane resolution, potential partial-volume effects, and sensitivity of parameter estimation to AIF selection and deconvolution methods. Fourth, habitat-based analysis currently lacks direct pathological or genomic validation, which would enhance interpretability. Finally, the present study focused primarily on methodological comparison of habitat strategies rather than deployment-ready clinical prediction; thus, discrimination was prioritized over probability calibration, which should be addressed in future studies.

## Conclusion

This study developed an MRI-based model integrating multiparametric radiomics and spatial habitat features for identifying diffuse glioma phenotypes associated with integrated histologic-molecular risk. The findings suggest that imaging-derived features may provide complementary information for non-invasive risk assessment of diffuse gliomas. Future studies incorporating radiomics, genomic profiles, treatment response, and survival outcomes may help refine individualized risk assessment and support personalized glioma management.

## Supplementary Information

Below is the link to the electronic supplementary material.

Supplementary Material 1

## Data Availability

The datasets generated during and/or analysed during the current study are available from the corresponding author on reasonable request.

## Abbreviations

AIFs: Arterial input functions
AUC: Area under the receiver operating characteristic curve
CBF: Cerebral blood flow
CI: Confidence intervals
CNS: Central nervous system
DSC-MRI: Dynamic susceptibility contrast MRI
EH: Edema habitats
GLCM: Gray-level co-occurrence matrix
GLRLM: Gray-level run-length matrix
GLSZM: Gray-level size-zone matrix
GLDM: Gray-level dependence matrix
MRI: Magnetic resonance imaging
MTT: Mean transit time
NGTDM: Neighborhood gray-tone difference matrix
RFE: recursive feature elimination
ROIs: The regions of interest
SVD: Singular value decomposition
T1W: T1-weighted
T1C: Contrast-enhanced T1-weighted
T2W: T2-weighted
T2FLAIR: T2 fluid-attenuated inversion recovery
TH: Tumor habitats
VEGF: Vascular endothelial growth factor
WE: Whole edema
WT: Whole tumor
