# Supplementary material for: Advancing precision risk stratification in adult diffuse gliomas through DSC-MRI-based habitat analysis
Source: BMC Med Imaging. 2026 Jun 23;26:440. doi: 10.1186/s12880-026-02509-7 (PMC13548655; doi:10.1186/s12880-026-02509-7)
Supplement: Supplementary file 1 [file 12880_2026_2509_MOESM1_ESM.docx]

**Supplementary Materials for:**

**1. Supplementary Figure 1.** Patient selection flowchart.

**2. Supplementary Figure 2.** Flowchart of the classification for high - and low - risk molecular subtypes of adult diffuse glioma in this study(1). IDH, Isocitrate Dehydrogenase; TERT, Telomerase Reverse Transcriptase; EGFR, Epidermal Growth Factor Receptor; CDKN2A/B, Cyclin - Dependent Kinase Inhibitor 2A/2B; co-del, co-deletion; hom-del, homozygous deletion.

**3. Supplementary Table 1.** Operational definition and stratification criteria for "high-risk" and "low-risk" molecular subtypes based on the 2021 WHO Classification of Central Nervous System Tumors (CNS5).

| **Molecular Risk Stratification** | **Integrated Diagnosis (WHO CNS5 Criteria)** | **Key Molecular & Histological Alterations** | **Clinical/Biological Rationale** |
| --- | --- | --- | --- |
| **High-Risk Subtype** | Astrocytoma, IDH-wildtype | *IDH1/2* wildtype (with *TERT* promoter mutation, *EGFR* amplification, and/or +7/-10 chromosome copy number changes) | Represents highly aggressive glioblastoma-like behavior, even in histologically lower-grade tumors. |
|  | Astrocytoma, IDH-mutant (Grade 4) | *IDH1/2* mutation AND histologically Grade 4 (microvascular proliferation or necrosis) | High-grade malignancy with accelerated progression and poor prognosis. |
|  | Astrocytoma, IDH-mutant (with *CDKN2A/B* deletion) | *IDH1/2* mutation AND *CDKN2A/B* homozygous deletion (regardless of histological grade) | *CDKN2A/B* homozygous deletion is a molecular marker that automatically dictates a WHO Grade 4 designation. |
| **Low-Risk Subtype** | Astrocytoma, IDH-mutant (Grade 2 or 3) | *IDH1/2* mutation, *ATRX* loss/p53 mutation, WITHOUT *CDKN2A/B* homozygous deletion | Indolent to moderately progressive course; significantly better survival compared to IDH-wildtype. |
|  | Oligodendroglioma, IDH-mutant and 1p/19q-codeleted | *IDH1/2* mutation AND concomitant *1p/19q* codeletion (Grade 2 or 3) | Highly favorable prognosis, associated with robust responsiveness to alkylating chemotherapy and radiation. |

**4. Supplementary Figure 3. (a)** Determination of the optimal number of clusters and habitat visualization based on the Calinski-Harabasz index. The red dashed line indicates the optimal value at which the index begins to decline. Three clusters were identified as optimal for both tumor and peritumoral edema regions. **(b)** Habitat maps of the tumor and peritumoral edema regions based on the clustering results.

**5. Supplementary Table 2.** MRI scanning parameters of brain tumor.

| Parameter/Sequence | 3D TIW | 3D TIW +C | 3D T2FLAIR | T2W | DSC-MRI |
| --- | --- | --- | --- | --- | --- |
| TR (ms) | 7.2 | 7.2 | 6000 | 5385 | 1801 |
| TE (ms) | 3.1 | 3.1 | 430 | 95 | 33.3 |
| Flip angle | 10° | 10° | 56° | 180° | 90° |
| Slice thickness (mm) | 1.0 | 1.0 | 1.0 | 5.0 | 5.0 |
| Slice number | 256 | 256 | 256 | 23 | 23 |
| FOV (mm2) | 240×256 | 240×256 | 240×256 | 200×230 | 230×230 |

**6. DSC-MRI Data Processing and Analysis**

The post-processing of dynamic susceptibility contrast MRI (DSC-MRI) data was performed using a customized pipeline in MATLAB (R2021a, MathWorks, Natick, MA, USA). The workflow was designed in accordance with the recommendations from the Quantitative Imaging Biomarkers Alliance (QIBA) profile for DSC-MRI where applicable(2). The detailed steps are as follows:

**6.1.Data Preprocessing:**

Raw data in NIfTI format were first corrected for orientation, including axis flipping and mirroring, to ensure spatial consistency. The baseline signal intensity (S₀) was calculated as the mean signal across the first five time points of the series.

**6.2. ΔR2* Quantification and T1-Leakage Correction:**

Dynamic changes in the transverse relaxation rate (ΔR2*(t)) were calculated from the signal intensity time curve (S(t)) using the standard steady-state gadolinium approximation model:

$$\Delta R2^{*}(t)=-\frac{1}{TE}\cdot\ln(\frac{SI(t)}{SI_{0}+\varepsilon})$$

where TE = 33.3 ms is the echo time of the acquisition sequence . To avoid numerical instability in voxels with very high signal dropout, values of S(t) greater than 0.95·S₀ were clamped to 0.95·S₀ prior to log transformation. The calculated ΔR2*(t) values were truncated to a physiologically plausible range of 0–50 s⁻¹.

Given the potential for contrast agent extravasation and T1 leakage effects in gliomas, a leakage correction algorithm was applied. We employed the widely adopted Boxerman-Schmainda-Weisskoff (BSW) model to estimate and correct for T1-induced signal contributions. This method fits a bi-exponential model to the ΔR2(t) curve to isolate and remove the leakage component, yielding a corrected ΔR2(t) curve used for all subsequent perfusion parameter calculation.

**6.3 Arterial Input Function (AIF) Detection**

AIF identification was performed at the target imaging slice (layer 9) by analyzing the signal slope during the early time window (frames 4–7) to locate candidate vascular regions. Morphological top-hat filtering (structuring element: circular disk with 5-pixel radius) was applied to enhance local features. Final AIF curves were selected using an interactive ROI (5 × 5 pixels) defined by the user and were validated based on peak signal intensity.

**6.4 Perfusion Parameter Calculation**

Tissue residue functions were computed via singular value decomposition (SVD) deconvolution, using a cutoff threshold of 15% of the maximum singular value to suppress high-frequency noise.

**Cerebral blood flow (CBF)** was estimated from the peak of the residue function.

**Cerebral blood volume (CBV)** : was calculated as the ratio of the integrals of the tissue and arterial ΔR2*(t) curves:

$$CBV =\frac{\left( \int\Delta R2*ₜᵢₛₛᵤₑ\left( t \right)\mathrm{dt} \right)}{\left( \int\Delta R2*ₐᵣₜₑᵣᵢₐₗ\left( t \right)\mathrm{dt} \right)}$$

**Mean transit time (MTT)** was derived as MTT = CBV/CBF. Physiologically implausible MTT values (<0 or >15 s) were excluded from further analysis.

**7. Supplementary Table 3.** Performance of multiple models for predicting high-risk molecular subtypes of adult diffuse glioma.

| Primary tumor regions | | | Model | **AUC(95%CI)** | **Se** | **Sp** | **PPV** | **NPV** | **Ac** | **Pre** | **Rec** | **F1** |
| --- | --- | --- | --- | --- | --- | --- | --- | --- | --- | --- | --- | --- |
| Tumor | Whole | | T1C+FLAIR | 0.871 (0.758, 0.944) | 0.889 | 0.800 | 0.821 | 0.800 | 0.814 | 0.810 | 0.792 | 0.799 |
|  |  |  | CBF+MTT | 0.902 (0.797, 0.964) | 0.889 | 0.833 | 0.914 | 0.833 | 0.881 | 0.874 | 0.879 | 0.876 |
|  |  |  | CBF+MTT+T1C+FLAIR | 0.931 (0.833, 0.980) | 0.889 | 0.840 | 0.941 | 0.840 | 0.898 | 0.891 | 0.901 | 0.895 |
|  | Habitat | | T1C+FLAIR | 0.895 (0.787, 0.960) | 0.861 | 0.773 | 0.838 | 0.773 | 0.814 | 0.805 | 0.800 | 0.802 |
|  |  |  | CBF+MTT | 0.944 (0.852, 0.987) | 0.889 | 0.818 | 0.865 | 0.818 | 0.847 | 0.842 | 0.836 | 0.838 |
|  |  |  | CBF+MTT+T1C+FLAIR | 0.937 (0.842, 0.984) | 0.833 | 0.760 | 0.882 | 0.760 | 0.831 | 0.821 | 0.830 | 0.824 |
| Edema | | Whole | T1C+FLAIR | 0.835 (0.715, 0.919) | 0.750 | 0.625 | 0.771 | 0.625 | 0.712 | 0.698 | 0.701 | 0.699 |
|  |  |  | CBF+MTT | 0.808 (0.684, 0.899) | 0.806 | 0.720 | 0.853 | 0.720 | 0.797 | 0.786 | 0.794 | 0.789 |
|  |  |  | CBF+MTT+T1C+FLAIR | 0.832 (0.711, 0.916) | 0.917 | 0.833 | 0.805 | 0.833 | 0.814 | 0.819 | 0.784 | 0.794 |
|  |  | Habitat | T1C+FLAIR | 0.819 (0.698, 0.907) | 0.833 | 0.667 | 0.732 | 0.667 | 0.712 | 0.699 | 0.678 | 0.682 |
|  |  |  | CBF+MTT | 0.932 (0.835, 0.981) | 0.889 | 0.826 | 0.889 | 0.826 | 0.864 | 0.857 | 0.857 | 0.857 |
|  |  |  | CBF+MTT+T1C+FLAIR | 0.905 (0.800, 0.966) | 0.861 | 0.762 | 0.816 | 0.762 | 0.797 | 0.789 | 0.778 | 0.783 |
| Tumor+Edema | | Whole | T1C+FLAIR | 0.856 (0.740,0.934) | 0.861 | 0.773 | 0.838 | 0.773 | 0.814 | 0.805 | 0.800 | 0.802 |
|  |  |  | CBF+MTT | 0.894 (0.786, 0.959) | 0.861 | 0.808 | 0.939 | 0.808 | 0.881 | 0.874 | 0.887 | 0.878 |
|  |  |  | CBF+MTT+T1C+FLAIR | 0.908 (0.804, 0.968) | 0.889 | 0.818 | 0.865 | 0.818 | 0.847 | 0.842 | 0.836 | 0.838 |
|  |  | Habitat | T1C+FLAIR | 0.885 (0.776, 0.953) | 0.889 | 0.826 | 0.889 | 0.826 | 0.864 | 0.857 | 0.857 | 0.857 |
|  |  |  | CBF+MTT | 0.949 (0.858, 0.989) | 0.944 | 0.905 | 0.895 | 0.905 | 0.898 | 0.900 | 0.885 | 0.891 |
|  |  |  | CBF+MTT+T1C+FLAIR | 0.948 (0.857, 0.989) | 0.944 | 0.900 | 0.872 | 0.900 | 0.881 | 0.886 | 0.864 | 0.872 |

CBF, cerebral blood flow; MTT, mean transit time; AUC, the area under the receiver operating characteristic curve; Se, Sensitivity; Sp, Specificity; PPV, Positive predictive value; NPV, Negative predictive value; Ac, Accuracy; Pre, Precision; Rec, Recall; F1, F1-score.

**8. Supplementary Figure 4.** Comparison of the area under the ROC curve between the whole region model and the habitat model. Statistical significance for the paired ROC curve comparisons was determined using DeLong's test, with p values adjusted for multiple comparisons using the Benjamini-Hochberg false discovery rate (FDR) correction. **p* < 0.05, ***p* < 0.01, ****p* < 0.001, ns = not statistically significant. AUC, the area under the receiver operating characteristic curve.

**9. Supplementary Figure 5.** Decision curve analysis (DCA). (a)The proposed habitat-based hemodynamic model (red curve) demonstrates the highest net benefit across a wide range of threshold probabilities, significantly outperforming the conventional whole-tumor anatomical model (blue curve). (b) ROI comparison: The combined Tumor+Edema model achieves the highest net benefit compared to single-region models, validating the prognostic value of the peritumoral edema region. (c) Modality comparison: Models derived solely from hemodynamic features (CBF+MTT) exhibit robust net benefit comparable to multimodal fusion models.

**10. Supplementary Figure 6. Data training workflow diagram.**

**Reference**

1.	Yang X, Niu W, Wu K, et al. Diffusion kurtosis imaging-based habitat analysis identifies high-risk molecular subtypes and heterogeneity matching in diffuse gliomas. Annals of clinical and translational neurology 2024;11(8):2073-2087.

2.	Shiroishi MS, Erickson BJ, Hu LS, et al. The QIBA Profile for Dynamic Susceptibility Contrast MRI Quantitative Imaging Biomarkers for Assessing Gliomas. Radiology 2024;313(3):e232555.
